# Searching for Beta-Haemolysin *hlb* Gene in *Staphylococcus pseudintermedius* with Species-Specific Primers

**DOI:** 10.1007/s00284-016-1038-4

**Published:** 2016-04-16

**Authors:** Wioletta Kmieciak, Eligia M. Szewczyk, Marcin Ciszewski

**Affiliations:** Department of Pharmaceutical Microbiology and Microbiological Diagnostics, Medical University of Łódź, 137 Pomorska St., 90-235 Lodz, Poland

## Abstract

The paper presents an analysis of 51 *Staphylococcus pseudintermedius* clinically isolated strains from humans and from animals. *Staphylococcus pseudintermedius* strains’ ability to produce β-haemolysin was evaluated with phenotypic methods (hot–cold effect, reverse CAMP test). In order to determine the *hlb* gene presence (coding for β-haemolysin) in a genomic DNA, PCR reactions were conducted with two different pairs of primers: one described in the literature for *Staphylococcus aureus* and recommended for analysing SIG group staphylococci and newly designed one in CLC Main Workbench software. Only reactions with newly designed primers resulted in product amplification, the presence of which was fully compatible with the results of phenotypic β-haemolysin test. Negative results for *S. aureus* and *S. intermedius* reference ATCC strains suggest that after further analysis the fragment of *hlb* gene amplified with primers described in this study might be included in the process of *S. pseudintermedius* strains identification.

## Introduction

*Staphylococcus pseudintermedius* belongs to the coagulase-positive staphylococci and together with *Staphylococcus intermedius* and *Staphylococcus delphini* constitutes SIG group (“*Staphylococcus intermedius* group”). *S. pseudintermedius* colonizes skin and mucosal membranes of animals, notably dogs and cats, and constitutes their opportunistic pathogen [5]. This species is prevalent in a veterinary hospital environment [15, 25, 32], which might be connected with the fact that people having frequent contact with animals (especially pets’ owners or veterinary personnel) usually become carriers of this species of bacteria [1, 13, 18, 19]. Human infections due to *S. pseudintermedius* occur usually in immunocompromised patients; however, their frequency has been still increasing [29, 31]. Infections in humans, such as catheter-borne bacteremia [3], sinusitis [27], infective endocarditis [20], non-hospital pneumonia [17] and wound infection after bone marrow transplantation [23] have already been noted. Further increase in the number of infections is highly possible, due to the fact that *S. pseudintermedius* is well equipped with various virulence factors i.e. coagulase, protease, enterotoxins, SIET exfoliative toxin, Luk-I leukotoxin and haemolysins (mainly β, but some strains also δ and α) [2, 8, 11].

The haemolysin type that has been most precisely described in the literature is staphylococcal β-haemolysin produced by *Staphylococcus aureus* and it constitutes a benchmark to haemolysin studies in other species [7]. In *S. pseudintermedius*, similarly to *S. intermedius*, β-haemolysin is considered to be produced constitutively [24]. β-haemolysin (sphingomyelinase) has a unique mechanism of action, such that it hydrolyses one of cell membrane lipids (sphingomyelin) to ceramides and phosphorylcholine, leading to cell lysis due to cell membrane destabilization [16]. Additionally, it stimulates the process of biofilm formation in vivo [14], exhibits cytolytic activity against human monocytes and macrophages [30], and it inhibits chemotaxis [28].

The activity of β-haemolysin is usually tested using sheep erythrocytes due to significant amount of sphingomyelin in their cytoplasmic membranes. The haemolytic effect is reinforced by lowering the incubation temperature, which prompts the characteristic hot–cold effect [26]. Co-haemolysis in reverse CAMP test and other CAMP-like tests is another method to test for the presence of β-haemolysin [21]. Molecular analyses detecting *hlb* gene, coding for β-haemolysin are becoming also more and more frequently used [10, 11].

## Methods

### Bacterial Strains

51 clinical strains of *Staphylococcus pseudintermedius* (13 obtained from humans and 38 from animals, mainly from dogs) were analysed, as well as 6 clinical strains of *Staphylococcus epidermidis* isolated from humans used as a negative control (this species does not produce β-haemolysin). All the tested strains were obtained from hospital and veterinary laboratories in Lodz, Poland. Strains were identified with MALDI-TOF system (Matrix-Assisted Laser Desorption/Ionization—Time of Flight Analysis) [4] and with genotypic method previously described by Sasaki et al. [22]. *Staphylococcus aureus* ATCC^®^ 25923 and *S. intermedius* ATCC^®^ 29663 reference strains were obtained from ATCC (LGC Standards) collection.

### Hot–Cold Effect

Analysed strains were incubated on a 5 % sheep blood agar at 37 °C for 24 h. Afterwards, the haemolysis effect was tested for. Subsequently, they were incubated at 4 °C for the next 16 h and analysed again. The enlargement of haemolysis zone around bacterial colonies after incubation at 4 °C (“double” haemolysis) was considered as a positive result.

### Reverse CAMP Test

In the middle of the 5 % sheep blood agar, reference strain of *Streptococcus agalactiae* (producing CAMP factor) was inoculated. Analysed strains were inoculated perpendicularly to the reference strain. Afterwards, the culture was incubated in 37 °C for 24 h. An enlarged haemolysis zone near the reference *S. agalactiae* strain (“arrowhead”) was considered as a positive result.

### DNA Isolation

Genomic DNA isolation was performed from overnight bacterial culture according to Genomic Mini AX BACTERIA SPIN (A&A Biotechnology) protocol.

### PCR Reactions

In order to determine the *hlb* gene presence in genomic DNA, PCR reactions were conducted with 2 different pairs of primers: one recommended in the literature [11] and another one, which was newly designed in the CLC Main Workbench 7.6 (QIAGEN) software, basing on *S. pseudintermedius* ED99 complete genome deposited in Genbank (NC_017568.1). PCR reaction temperature profile was as follows: initial denaturation 2:30 min. −94 °C, 30 cycles (denaturation 0:30 min. −94 °C, annealing 0:30 min. −56 °C, elongation 1:00 min. −72 °C) and final elongation 10:00 min. −72 °C. Primer sequences and expected amplicon sizes are presented in Table 1.

**Table 1:** Primers used in this study

Primers for *hlb* gene	Sequence	Amplicon size
Described in the literature [11]	5′-GTGCACTTACTGACAATAGTGC-3′	309 bp
	5′-GTTGATGAGTAGCTACCTTCAGT-3′	
Newly designed	5′-GACGAAAATCAAGCGGAA-3′	734 bp
	5′-TCTAAATACTCTGGCGCAC-3′	

### Agarose Gel Electrophoresis

PCR products were separated during electrophoresis in 1 % agarose gel (TAE buffer, 70 V, 60 min.).

### Statistical Analysis

Statistical analysis was performed using STATISTICA 10 software (Statsoft).

## Results

The results of phenotypic and genotypic analyses for *S. pseudintermedius* strains are shown in Table 2.

**Table 2:** The results of phenotypic and genotypic tests for *S. pseudintermedius* strains, evaluating the ability to produce β-haemolysin and the presence of *hlb* gene

Strains	Hot–cold effect	Reverse CAMP	*hlb* (literature primers)	*hlb* (new primers)
SPI 188, SPI 237	+	+	+	+
SPI 150, SPI 187, SPI 197, SPI 211, SPI 215, SPI 216, SPI 222, SPI 227, SPI 228, SPI 230, SPI 273, SPI 286, SPI 305, SPI 324, SPI 378, SPI 391, SPI 397, SPI 398, SPI 399, SPI 404, SPI 418, SPI 434, SPI 442, SPI 526, SPI 639, SPI 671, SPI 699, SPI 796, SPI-X3	+	+	−	+
SPI 185, SPI 186, SPI 205, SPI 206, SPI 207, SPI 285, SPI 302, SPI 325, SPI 330, SPI 340, SPI 344, SPI 357, SPI 369, SPI 370, SPI 373, SPI 443, SPI 445, SPI 525	+	−	−	+
SPI 323	−	−	−	−

**Fig. 1:**
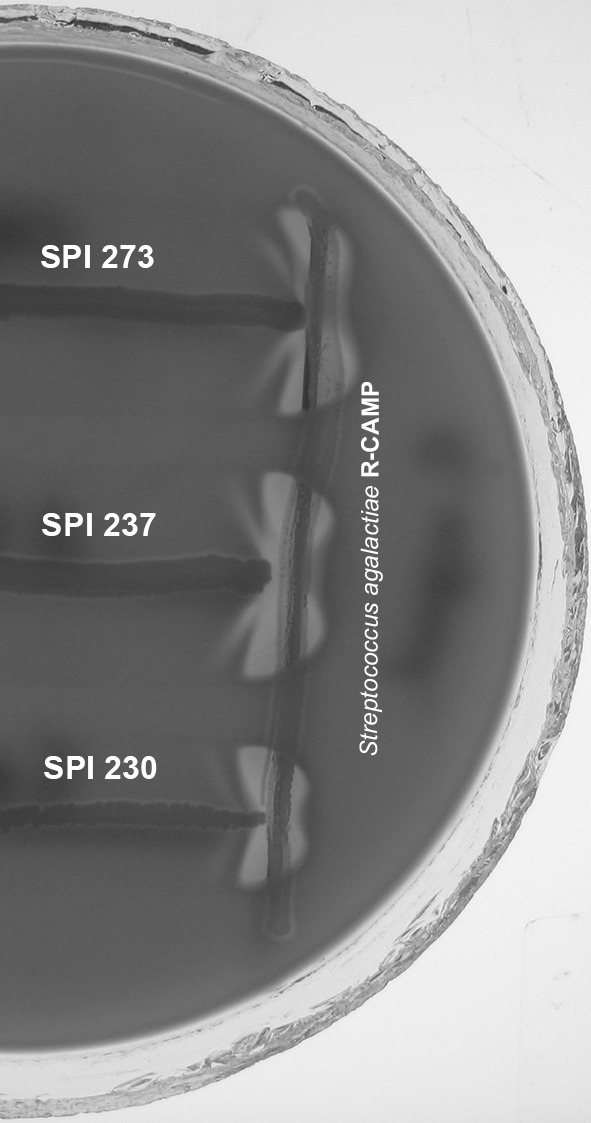
Reverse CAMP test results of the selected *S. pseudintermedius* strains

β-haemolysin was phenotypically detected (hot–cold effect and reverse CAMP test) in 61 % of analysed *S. pseudintermedius* and none of *S. epidermidis* negative control strains. One of *S. pseudintermedius* strains did not produce β-haemolysin. In 35 % of *S. pseudintermedius* strains, β-haemolysin production was detected only by hot–cold effect, whereas the reverse CAMP test was negative (Fig. 1).

The presence of *hlb* gene, in a PCR reaction with primers described in the literature [11], was confirmed only in 2 (4 %) *S. pseudintermedius* strains, whereas in the reaction with newly designed primers—in 50 (98 %) analysed *S. pseudintermedius* strains. The absence of *hlb* gene was detected only in SPI 323 animal strain which was also negative in phenotypic β-haemolysin test.

**Fig. 2:**
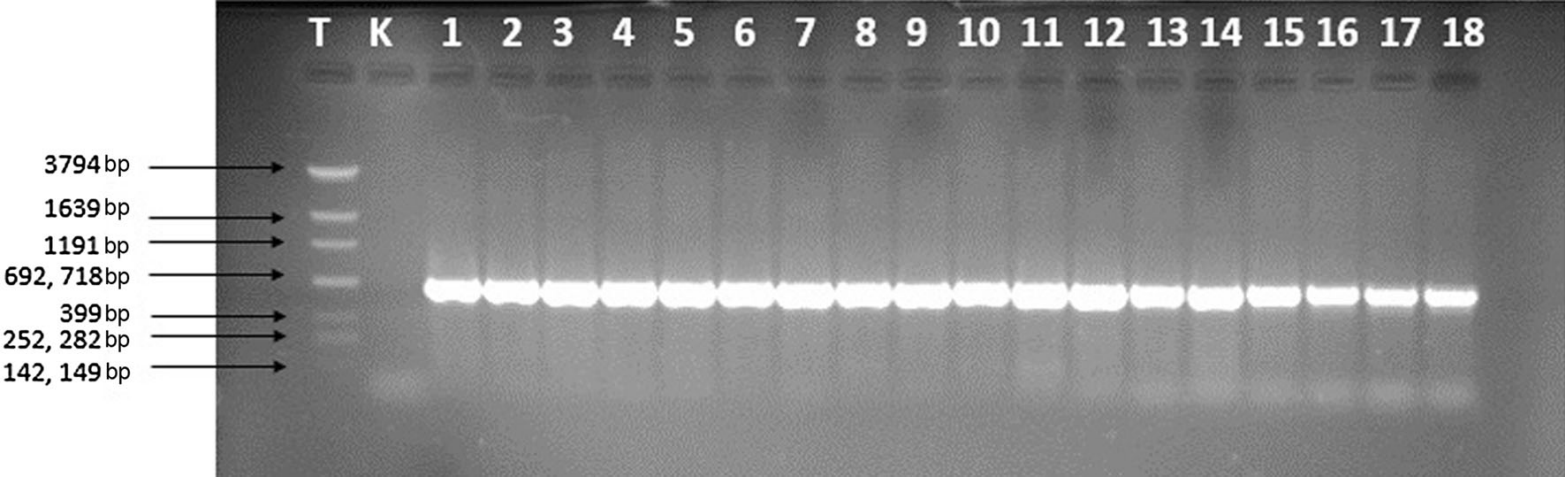
Agarose gel electrophoresis of the selected PCR products after reaction with the newly designed primers (T—DNA Marker DraMix, K—negative control, 1–18—*S. pseudintermedius* strains SPI 150—SPI 391)

The *hlb* gene was detected in none of *S. epidermidis* control strains, nor in the *S. aureus* ATCC^®^ 25923 and *S. intermedius* ATCC^®^ 29663 reference strains. Results were negative in PCR reactions when both the described in the literature and the newly designed primers were used (Fig. 2).

The statistical analysis based on the *χ*^2^ test showed that the relationship between hot–cold effect and *hlb* gene presence in the PCR reaction with the newly designed primers was statistically significant (*P* = 0.00000).

## Discussion

*S. pseudintermedius* strains are thought to constitutively produce β-haemolysin and rarely δ-haemolysin [24]. Apart from classic phenotypic tests (hot–cold effect, reverse CAMP test), molecular analyses of β-haemolysin based on *hlb* gene searching are also used [6, 9, 11, 21].

PCR reactions conducted in this study with primers described by Gharsa et al. [11] showed positive results only in 2 (4 %) out of 51 analysed *S. pseudintermedius* strains. This contradicts previous results showing common phenotypic demonstration of β-haemolysin presence, as well as previously described genotypic studies proving *hlb* gene presence in strains able to produce β-haemolysin [9, 11, 12].

On the basis of the *S. pseudintermedius* ED99 complete genome deposited in Genbank, we designed a new pair of primers for *hlb* gene, which enable the analysis of *S. pseudintermedius* strains. PCR searching results completely confirmed phenotypic hot–cold test outcome, which proved to be more reliable than the reverse CAMP test.

The results described in this paper contest previous studies on the possibility of searching for *S. pseudintermedius* virulence genes using *hlb* primers described for *S. aureus* [11], because they seem to be inadequate for *S. pseudintermedius* strains. This result also contests the credibility of previously published analyses of bacterial strains from SIG group. Primers proposed in this study for searching for β-haemolysin *hlb* gene in *S. pseudintermedius* seem to be much more accurate in the detection of this virulence factor in bacterial strains of this species. Preliminary studies on the newly designed primers showed also negative *hlb* searching results for *S. aureus* and *S. intermedius* reference ATCC strains. This suggests that after further analysis, the fragment of *hlb* gene amplified with primers described in this study might be included in the process of *S. pseudintermedius* strains identification. That would be extraordinarily desirable because of numerous difficulties in the differentiation among the species of the SIG group.
